# Molecular docking analysis of protein filamin-A with thioazo compounds

**DOI:** 10.6026/97320630019099

**Published:** 2023-01-01

**Authors:** Sudarshan Satish, Gayathri Rengasamy, Surya Sekaran, Kavitha Sankaran, Vishnu Priya Veeraraghavan, Rajalakshmanan Eswaramoorthy

**Affiliations:** 1 Department of Biochemistry, Saveetha Dental College and Hospitals, Saveetha Institute of Medical and Technical Sciences, Saveetha University, Chennai-600077; 2 Department of Biomaterials (Green lab), Saveetha Dental College and Hospital, Saveetha Institute of Medical and Technical Science (SIMATS), Saveetha University, Chennai-600077

**Keywords:** Oral cancer, thio azo derivatives, protein filamin-a, drug discovery

## Abstract

It is of interest to document the molecular docking analysis of protein Filamin-A with thioazo compounds. The compounds 1, 3, 5, and 6 showed best molecular docking interaction as compared to the drug doxorubicin. Among the selected ligands (1-6), compound 3 shows better interaction score than doxorubicin and follows Lipinski's rule of five. Hence, it could be considered as a potential lead molecule for inhibiting protein filamin A in the treatment of oral cancer.

## Background:

Protein filamin A is commonly expressed in oral Cancer. Due to its dual mechanism, it promotes cancer if present in cytoplasm and suppresses the tumor if present in plasma membrane. Development of drugs to target FLN-A cause cleavage and subsequent localization to the nucleus, this could be a new and potent field of research in treating cancer. Protein Filamin A is the first actin filament cross linking protein or gelation factor to be found in non-muscle cells for more than 90 binding proteins (Fln b and c) the products of separate genes list the partners implicated in cell adhesion and migration. The other partners are human FLN gene mutations induce a wide variety of cell and tissue abnormalities due to the extensive array of related proteins [1]. The role of FLNa in cell migration and adhesion are the main topics. Two 280kDa subunits make up FLNa which self assembles into a 160 nm semi flexible stand. Each FLN subunit has 24 repeating plated sheet units at the end of its N terminal spectrin related actin binding domain. The repeats are divided into rod1(repeats 1-5) rod 2 (repeats 10-23) and self-association domain by two intervening Calpain sensitive hinges (IgFLN) each of which is made up of seven runs of ( AG) strands 9, 10IgFLNa 24 the most C terminal repeat, mediates [2]. The C terminal repeat IgFLNa24 facilitates dimerization and gives dimeric molecules a V shape which causes F actin to branch perpendicularly. The F actin binding domain Rod2 on the other hand associates with partner proteins and most partner interactions occur within the rod 2 domain [3]. Binding and positioning of multiple partners in close proximities on rod 2 facilitates signal transduction of FLNa enriched sites in cell. The most prevalent and extensively distributed number of filamin proteins is FLNa which is encoded on the X chromosomes. On human chromosome 3, FLNb a non-muscle is encoded [4]. The FLN gene is found in chromosome 7 and is mostly expressed in smooth, striated similarities and differences between 56, 57 FLN isoform structures, expression levels and localization characteristics [5]. Therefore, it is of interest to document the molecular docking analysis of protein Filamin-A with thioazo compounds.

## Material and Methods:

## Ligand preparation:

**Figure 1:**
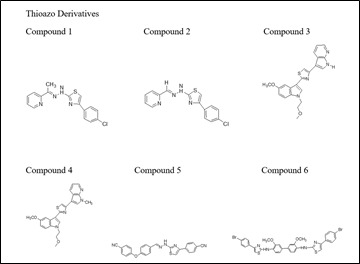
2D Structure of the Thioazo Compounds (1-6)

The 2D chemical structures of the thioazo compounds (1-6) were prepared using ChemOffice Suite 16.0 (Figure 1). During the optimization method, the software Chem3D was employed and all parameters were selected in order to achieve a stable structure with the least amount of energy. The structural optimization approach was used to estimate the global lowest energy of the title chemical. Each molecule's 3D coordinates (PDB) were determined using optimized structure.

## Protein preparation:

**Figure 2:**
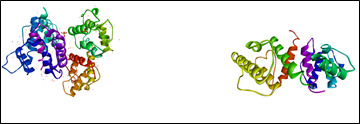
3D structure of the protein Filamin A from *Homo sapiens*

The 3D structure of Protein Filamin A from *Homo sapiens* was retrieved from the protein data bank (PDB Id: 3HOP) and was prepared in accordance with standard protocol (Figure 2). Water molecules, other hetero atoms, co-crystallized ligands were removed, and the protein were prepared by adding polar hydrogens and kollman charges with Auto Prep.

## Molecular docking:

**Figure 3:**
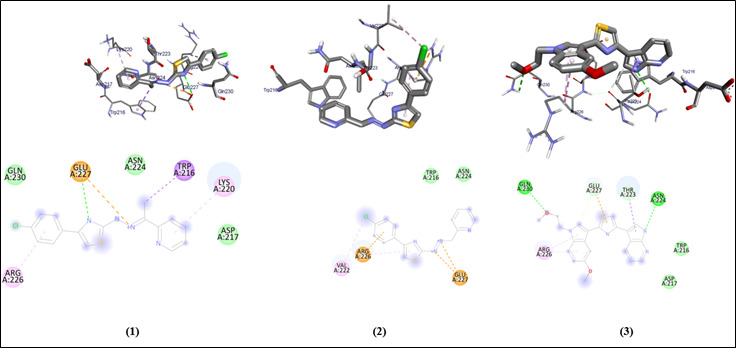
Molecular docking analysis of compounds (1-3) against Protein filamin A of *Homo sapiens*

**Figure 4:**
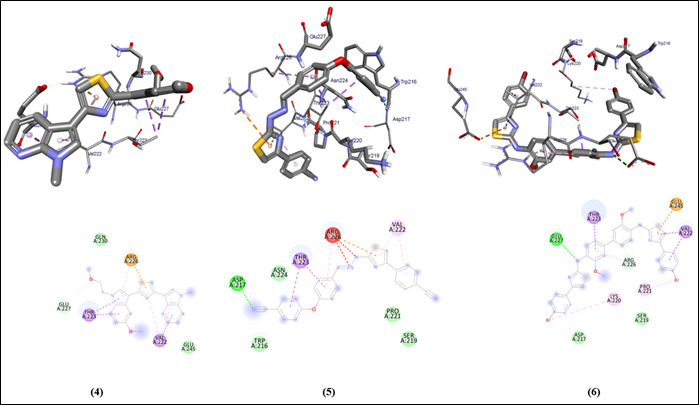
Molecular docking analysis of compounds (4-6) against Protein filamin A of *Homo sapiens*

The graphical user interface Auto Dock vina was used for Ligand-Protein docking interactions (Figure 3 and Figure 4). Auto Dock Tools (ADT), a free visual user interface (GUI) for the AutoDock Vina software, was used for the molecular docking research. In order to dock the compounds (1-6) against the protein's active site, AutoDockVina was employed with a grid point center spacing of 34.423, -24.624, -32.632 along the x, y, z axis respectively. The dimensions (Angstrom) of the grid box are 17.959, 15.780, and 16.467 that point in the x, y, and z directions, respectively. For eachligand, nine alternative conformations were created and ranked based on their binding energies using the AutoDockVina scoring functions. The post-docking evaluations were conducted using PyMOL and AutoDock Tools.

## ADMET Analysis:

The SwissADME and PRO-TOX II online servers were used for estimating the absorption, distribution, metabolism, and excretion and toxicity profiles. The SwissADME, a web tool from Swiss Institute of Bioinformatics (SIB) is used to convert the two-dimensional structures into their simplified molecular input line entry system (SMILES). The physicochemical properties (molar refractivity, topological polar surface area, number of hydrogen bond donors/ acceptors); pharmacokinetics properties (GI absorption, BBB permeation, P-gp substrate, cytochrome-P enzyme inhibition, skin permeation (log Kp)) which are critical parameters for prediction of the absorption and distribution of drugs within the body, and drug likeness (Lipinski's rule of five) were predicted using SwissADME. The toxicological endpoints (Hepatotoxicity, Carcinogenicity, Immunotoxicity, and Mutagenicity) and the level of toxicity (LD50, mg/Kg) are determined using the ProTox-II server.

## Statistical Analysis:

One way ANOVA was used for statistical analysis. The clinically proven drugs are used as a control and the results are compared. The significance of the results was found to be p< 0.05

## Results:

## Molecular docking interaction of thioazo compounds against Protein Filamin A of *Homo sapiens*:

All the compounds (1-6) are run against the target against Protein Filamin A of *Homo sapiens* and it shows the range between -4.8 to -6.6 (Table 1). The compounds 1, 3, 5, and 6 showed best docking interaction as compared to drug Doxorubicin (-5.5 kcal/mol).

## SwissADME and Lipinski’s rule of five:

The compounds show log Kp values between -3.59 to -6.28 cm/s (Table 2). The compounds (1-4) show high gastro intestinal absorption so it doesn’t need a carrier molecule, whereas, compounds 5, and 6 shows low GI absorption, so it needs a carrier molecule. All the compounds show no blood brain barrier permeability. Compounds (1-5) except compound 6, obey Lipinski's rule of five (Table 3).

## Toxicity profiling:

The compounds 1, 2, and 5 shows class 3 toxicity but none of the molecules are cytotoxic. Compounds 3 and 4 shows a similar LD50 value (3000mg/kg) and class 5 toxicity. Also, they are inactive in hepatotoxicity, carcinogenicity, immunotoxicity, and cytotoxicity (Table 4). Hence, compounds 3, and 4 can be used as a potential lead.

## Discussion:

Mouth and throat cancers are included in the category of oral cancer. On the tongue, the skin lining the mouth and gums, beneath the tongue, at the base of the tongue, and in the region of the throat towards the rear of the mouth, oral malignancies can grow [6]. About 53,000 new cases of oral cancer, or 3% of all malignancies diagnosed each year are oral cancer. More than twice as many men as women are affected by oral cancer, which most frequently affects adults over the age of 40 [7]. The majority of oral malignancies are linked to tobacco use, alcohol consumption (or both), or human papillomavirus infection (HPV) [8]. Doxorubicin is an excellent treatment option for various malignancies, but its practical application is constrained by the severe side effects that can occur at the maximum effective dose [9]. In a study, human hepatoma HepG2 cells were used to evaluate potential methods to increase the effectiveness of low-dose doxorubicin, including a metronomic schedule, which entails brief and repeated exposure to the anticancer medication, and the combination with the naturally occurring chemosensitizing sesquiterpenes -caryophyllene and -caryophyllene oxide [10]. A broad-spectrum anticancer agent known as paclitaxel was first isolated from a medicinal plant, specifically the bark of the yew tree Taxus brevifolia Nutt [11]. It belongs to a group of diterpene taxanes, which are presently the most widely used chemotherapeutic drugs for treating various cancers [12]. It possesses ovarian, lung, and breast cancer-fighting properties that have been clinically verified. This compound's low solubility, re-crystallization after dilution, and cosolvent-induced toxicity make use challenging. In certain situations, nanotechnology and nanoparticles offer several benefits over free pharmaceuticals, including increased drug half-life, decreased toxicity, and targeted and selective delivery [13]. Nanodrugs can accumulate in tissue, which may be related to increased permeability and retention as well. The compounds 1, 3, 5, and 6 show molecular docking interactions better than the clinically proven drugs Doxorubicin -5.5 kcal/mol [14]. All the molecules follow Lipinski's rule of 5 except compound 6 so it is ruled out. The log kp value of 3 and 5 are -5.2 and -5.4 respectively, which denotes they are skin absorbable. All the compounds except 5 and 6 have high gastrointestinal absorption [15]. All the compounds show a similar toxicity profile to Doxorubicin and Paclitaxel and they are nontoxic. The compound 3 is best among all the compounds [16].

## Conclusion:

Data shows that compound #3 shows better interaction than doxorubicin and paclitaxel and follows Lipinski's rule of 5. Hence, it could be considered as a potential lead molecule for inhibiting protein Filamin A in the treatment of cancer.

## Scope for future research:

The molecule has to be developed for future research. Compounds with functional groups similar to lead molecules have to be explored.

## Figures and Tables

**Table 1:** Molecular docking scores and residual amino acid interactions of Thioazo compounds (1-6) against Protein filamin A (FLNA) of *Homo sapiens* (PDB ID - 3HOP)

Ligands	Docking scores/Affinity (kcal/mol)	H-bond	Amino Acid Residual interactions	
			Hydrophobic/Pi-Cation	Van dar Waals
1	-5.6		Glu-227, Trp-216, Lys-220, Arg-226	Gln-230, Asn-224, Asp-217
2	-5.2		Arg-226, Val-222, Glu-227	Trp-216, Asn-224
3	-5.5	Gln-230, Asn-224	Glu-227, Thr-223, Arg-226	Asp-217, Trp-216
4	-4.8		Arg-226, Thr-223, Val-222, Glu-227	Glu-245, Gln-230
5	-6.6	Asp-217	Val-222, Arg-226, Thr-223	Trp-216, Pro-221, Ser-219, Asn-224
6	-5.9	Glu-227	Glu-245, Val-222, Pro-221, Lys-220, Thr-223, Arg-226	Ser-219, Asp-217
Doxorubicin	-5.5	Glu-245, Asp-249, Gln-193	Pro-221, Pro-250	Val-248, Thr-223
Paclitaxel	-4.5		Trp-216, Thr-223, Lys-220	Glu-227, Asn-224, Asp-212, Ser-215, Arg-226
Tamoxifen	-4.6		Asp-249, Pro-250, Pro-221, Val-222	Glu-245, Gln-193, Ser- 194, Ala-218, Val-248

**Table 2:** SwissADME values of selected thio azo compounds (1-6)

Compound	log Kp (cm/s)	GI absorption	BBB permeant	Pgp substrate	CYP1A2 inhibitor	CYP2C19 inhibitor	CYP2C9 inhibitor	CYP2D6 inhibitor	CYP3A4 inhibitor
1	-4.98	High	No	No	Yes	Yes	Yes	No	Yes
2	-5.05	High	No	No	Yes	Yes	Yes	No	No
3	-6.28	High	No	Yes	Yes	Yes	Yes	Yes	Yes
4	-6.4	High	No	Yes	Yes	Yes	Yes	Yes	Yes
5	-4.94	Low	No	No	No	Yes	Yes	No	Yes
6	-3.59	Low	No	No	No	No	No	No	No
Doxorubicin	-8.71	Low	No	Yes	No	No	No	No	No
Paclitaxel	-8.91	Low	No	Yes	No	No	No	No	No
Tamoxifen	-3.5	Low	No	Yes	No	Yes	No	Yes	No

**Table 3:** Lipinski and Veber rules of selected thioazo compounds (1-6)

Compound	MW	iLogP	HBD (nOHNH)	HBA (nON)	nrotb	MR	TPSA	Lipinski #violations	Bio availability score
Lipinski*	≤500	≤5	≤5	≤10	≤10	-	-		
Veber**	-	-	-	-	-	-	≤ 140		
1	328.82	2.65	1	3	4	92.64	78.41	0	0.55
2	314.79	2.26	1	3	4	87.83	78.41	0	0.55
3	404.48	3.47	1	4	6	116.59	93.2	0	0.55
4	418.51	3.84	0	4	6	121.49	82.34	0	0.55
5	421.47	3.4	1	5	6	120.97	122.33	0	0.55
6	720.5	6.24	2	4	9	181.57	124.78	2	0.17
Doxorubicin	543.52	2.16	6	12	5	132.66	206.07	3	0.17
Paclitaxel	853.91	4.51	4	14	15	218.96	221.29	2	0.17
Tamoxifen	371.51	4.64	0	2	8	119.72	12.47	1	0.55

**Table 4:** Toxicity profile of selected thioazo compounds (1-6)

			Toxicity				
Compound	^a^LD_50_ (mg/kg)	Class	HEPATOTOXICITY	CARCINOGENICITY	IMMUNOTOXICITY	MUTAGENICITY	CYTOTOXICITY
1	300mg/kg	3	Active	Inactive	Active	Inactive	Inactive
2	300mg/kg	3	Active	Inactive	Active	Inactive	Inactive
3	3000mg/kg	5	Inactive	Inactive	Active	Inactive	Inactive
4	3000mg/kg	5	Inactive	Inactive	Active	Inactive	Inactive
5	300mg/kg	3	Active	Active	Inactive	Active	Inactive
6	1000mg/kg	4	Active	Active	Active	Active	Inactive
Doxorubicin	205mg/kg	3	Inactive	Inactive	Active	Active	Active
Paclitaxel	134mg/kg	3	Inactive	Inactive	Active	Inactive	Active
Tamoxifen	1190mg/kg	4	Active	Inactive	Active	Inactive	Inactive
a LD50: lethal dose parameter
